# Variable Responses of Benthic Communities to Anomalously Warm Sea Temperatures on a High-Latitude Coral Reef

**DOI:** 10.1371/journal.pone.0113079

**Published:** 2014-11-26

**Authors:** Tom C. L. Bridge, Renata Ferrari, Mitch Bryson, Renae Hovey, Will F. Figueira, Stefan B. Williams, Oscar Pizarro, Alastair R. Harborne, Maria Byrne

**Affiliations:** 1 ARC Centre of Excellence for Coral Reef Studies, James Cook University, Townsville, QLD, Australia; 2 Australian Institute of Marine Science, PMB #3, Townsville MC, Townsville, QLD, Australia; 3 School of Biological Sciences, University of Sydney, Sydney, NSW, Australia; 4 Australian Centre for Field Robotics, School of Engineering, University of Sydney, Sydney, NSW, Australia; 5 School of Earth and Environment and Oceans Institute, University of Western Australia, Crawley, WA, Australia; 6 Marine Spatial Ecology Laboratory and ARC Centre of Excellence for Coral Reef Studies, School of Biological Sciences, The University of Queensland, Brisbane, QLD, Australia; 7 School of Anatomy, University of Sydney, Sydney, NSW, Australia; Dauphin Island Sea Lab, United States of America

## Abstract

High-latitude reefs support unique ecological communities occurring at the biogeographic boundaries between tropical and temperate marine ecosystems. Due to their lower ambient temperatures, they are regarded as potential refugia for tropical species shifting poleward due to rising sea temperatures. However, acute warming events can cause rapid shifts in the composition of high-latitude reef communities, including range contractions of temperate macroalgae and bleaching-induced mortality in corals. While bleaching has been reported on numerous high-latitude reefs, post-bleaching trajectories of benthic communities are poorly described. Consequently, the longer-term effects of thermal anomalies on high-latitude reefs are difficult to predict. Here, we use an autonomous underwater vehicle to conduct repeated surveys of three 625 m^2^ plots on a coral-dominated high-latitude reef in the Houtman Abrolhos Islands, Western Australia, over a four-year period spanning a large-magnitude thermal anomaly. Quantification of benthic communities revealed high coral cover (>70%, comprising three main morphospecies) prior to the bleaching event. Plating *Montipora* was most susceptible to bleaching, but in the plot where it was most abundant, coral cover did not change significantly because of post-bleaching increases in branching *Acropora*. In the other two plots, coral cover decreased while macroalgal cover increased markedly. Overall, coral cover declined from 73% to 59% over the course of the study, while macroalgal cover increased from 11% to 24%. The significant differences in impacts and post-bleaching trajectories among plots underline the importance of understanding the underlying causes of such variation to improve predictions of how climate change will affect reefs, especially at high-latitudes.

## Introduction

Increases in the frequency and intensity of acute and chronic disturbances are altering the structure and function of coral reef ecosystems globally [1]–[3]. Acute disturbances affecting reefs include warm-water thermal anomalies, which can cause abrupt shifts in the composition of coral reef assemblages [4]–[6]. Temperature-mediated bleaching occurs when the thermal tolerance of corals and their photosynthetic symbionts (zooxanthellae) is exceeded and can lead to widespread mortality [1], [2], [7]. In addition, surviving corals often exhibit sub-lethal effects following exposure to thermal stress, including increased susceptibility to disease and reductions in growth and fecundity that can inhibit coral recovery [7]–[9]. Reduced coral abundance, particularly in taxa with structurally complex morphologies, results in concomitant declines in other taxa dependent on coral-dominated reef ecosystems [10]–[14].

High-latitude, subtropical reefs provide a range of ecosystem goods and services, and have also been proposed as potential refugia for coral reef biodiversity from rising sea temperatures as tropical biota shift their distributions polewards [15]–[19]. Present-day high-latitude coral-assemblages are typically dominated by subtropical species with antitropical distributions, while species common on low-latitude tropical reefs are generally absent or rare [18], [20]. Corals on subtropical reefs generally exhibit lower bleaching thresholds than those at lower latitudes [21] because bleaching susceptibility is strongly correlated to thermal history [22], [23]. Although bleaching has been reported from numerous high-latitude reefs in recent years [24]–[26], the longer-term consequences of ocean warming on high latitude reefs are poorly understood.

Spatial variability in bleaching incidence is common due to a variety of factors including local-scale environmental conditions [27], [28], historical exposure to higher or more variable temperatures [29], [30], and differing susceptibilities among coral taxa [4], [31], [32]. Furthermore, proximal benthic communities often show remarkably different post-bleaching trajectories which cannot be easily attributed to environmental variability or management actions, such as protection of herbivorous fishes [33]–[35]. Intra-reef variability in response to bleaching might be particularly extensive on high-latitude reefs because of the mosaic of habitats present, such as coral- and kelp-dominated habitats found in close proximity [36], [37]. Despite increasing interest in the ecology of high-latitude reefs [18], [20], [38], [39], their temporal dynamics remain poorly described relative to tropical reef ecosystems. This lack of data is problematic because extrapolation of results from tropical reefs may not be appropriate. In addition to subtropical corals exhibiting lower bleaching thresholds, higher nutrient levels result in greater abundance of macroalgae on high-latitude reefs, which may potentially limit coral recovery after bleaching events [39], [40]. Even if tropical corals can disperse to higher latitudes, increased competition with macroalgae may inhibit their ability to establish viable populations [41]–[43]. Consequently, it is critical to understand the response of benthic communities on high-latitude reefs to disturbances such as acute thermal anomalies.

Here, we use repeat surveys conducted by an autonomous underwater vehicle (AUV) to quantify changes in benthic community composition over a four-year period (2010–2013) straddling a large-magnitude thermal anomaly in 2011 and smaller temperature anomalies in 2012 and 2013 at the Houtman Abrolhos Islands (HAI), a high-latitude reef system in Western Australia. The 2011 ‘marine heatwave’ was associated with strong La Niña conditions that caused extensive coral bleaching on Western Australian reefs [24], [26], [44], [45]. We quantify benthic community composition in three proximal 25×25 m (∼625 m^2^) ‘plots’ at the HAI. We aimed to (1) quantify the nature and magnitude of shifts in benthic community composition at intermediate but ecologically important spatial scales (hundreds of square metres); (2) identify differences in susceptibility among both coral and macroalgal taxa; and (3) determine how variability in the sensitivity to thermal stress among plots and taxa influenced the short term trajectories of benthic communities. Quantifying such spatial, temporal and taxonomic heterogeneity in the effects of temperature anomalies at the HAI can provide insight into the likely response of high-latitude reefs to rising sea temperatures and increasing frequency of thermal anomalies.

## Methods

No specific permissions were required for these activities and locations as no organisms were removed in the process of collecting this remote information. AUV surveys are conducted in collaboration with Fisheries Western Australia, the managing agency for the Houtman Abrolhos Islands, and are facilitated by Australia's Integrated Marine Observing system (IMOS) AUV Facility. The study did not involve endangered or protected species and was conducted at Geebank 28.81°S, 113.947°E.

### Study Site

**Figure 1:**
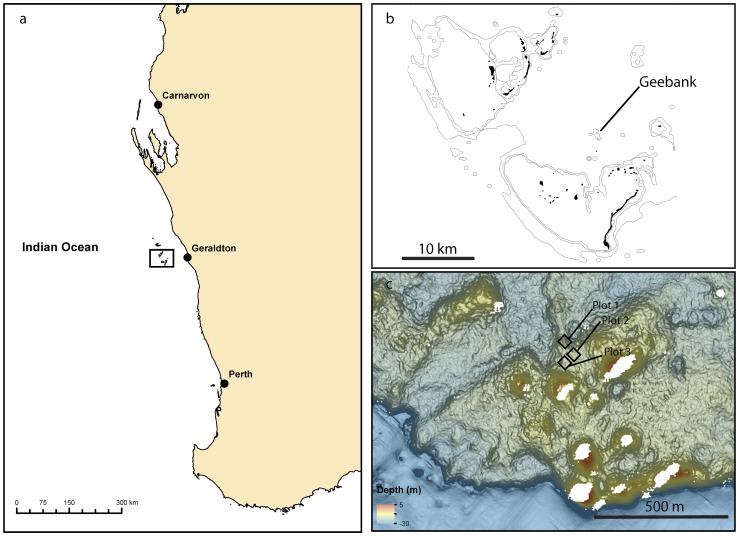
Location of the Houtman Abrolhos Islands, Western Australia (a); location of the study site Geebank between the Easter and Southern (Pelseart) island groups (b). Black squares indicate the location of replicate plots (c).

The HAI are a series of limestone outcrops on the edge of the continental shelf 60–80 km off the coast of Western Australia (Fig. 1a). Located between 28 and 29°S, they are among the highest-latitude coral reefs in the world [46]. Despite their location, 184 reef-building coral species have been recorded from the HAI [47]. Due to their unique location at the boundary of tropical and temperate zones, corals often occur in mixed communities with various macroalgae, including temperate taxa such as the kelp *Ecklonia radiata* [48]–[50]. Shifts in the latitudinal boundary of these two major biogeographic regions due to rising sea temperatures are therefore likely to be particularly apparent in the HAI [51]. We used the AUV *Sirius* to study coral assemblages at Geebank (28.81°S, 113.947°E), a submerged bank located between the Easter and Southern (Pelsaert) island groups of the HAI (Fig. 1b).

In the austral summer of 2010–11, Western Australia experienced an unprecedented ‘marine heatwave’ caused by an anomaly in the Leeuwin Current, a major poleward boundary current, associated with strong La Niña conditions [51], [52]. Average sea surface temperatures in February 2011 peaked at 3°C above long-term monthly averages along a large section of the Western Australian coast from Ningaloo Reef to Cape Leeuwin, an area spanning 12° of latitude [51]. In the (HAI), the average maximum summer (December to May) sea temperatures were at least 4.7°C above the previous 30-year average, and 4.3°C above the average for the previous 3 years (22.99°C±0.21°C based on HadISST) [16]. The marine heatwave resulted in the first documented mass bleaching event in the HAI, and also had detrimental effects on adjacent kelp communities [26], [44]. Additional smaller thermal anomalies occurred during the summer of 2012 and 2013 [53], although the effects of these events on benthic assemblages at the HAI have not been described.

### Data collection

The AUV *Sirius* collects geo-referenced stereo images from an altitude of 2 m with each stereo pair covering approximately 1.5×1.2 m of the seafloor [54]. The position of the AUV relative to the support ship is calculated using an Ultra Short Base Line (USBL) acoustic positioning system. This information is combined with the ship's GPS, the vehicle's on-board navigation sensors and the stereo imagery to determine the geo-referenced position of each individual image [55]. The position of each image across multiple year surveys was registered to an accuracy of 10 cm [56]. By using this geo-referencing system, *Sirius* is able to accurately survey the same sites across multiple years.

AUV surveys were conducted in three permanent 25×25 m ‘plots’ at a similar depth of ∼15–18 m in April of four consecutive years from 2010 to 2013 (Fig. 1c): before, during and two years after the bleaching event. Surveys were conducted at the same time of year (April) to allow comparison among years while minimizing the influence of seasonal variability of some taxa (e.g. macroalgae). Plots were spaced 50–100 m apart to ensure spatial independence and capture spatial heterogeneity within each site [57].The size and random placement of the plots increase the likelihood they are representative of the coral-dominated communities within the study area. All data collected by the AUV and used in this study are freely available through the Australian Ocean Data Network Portal at https://auv.aodn.org.au/auv/ and the Integrated Marine Observing System Data Portal http://imos.org.au/auv_data.html.

We selected a subset of 35 spatially balanced and randomly selected images from each plot for each year using a Generalized Random Tessellation Stratified (GRTS) sampling design in Matlab [58], resulting in a total of 420 images being analysed from 2010–2013. Thirty-five images were chosen based on a power analysis of changes in variance with increasing replication (up to 50 images) for one plot (Table S1). This analysis indicated little further reduction in residual standard error of percent cover for major benthic categories (i.e. table *Acropora*) above 30 images per plot. Power analysis also indicated minimal improvement in detectable effect sizes (given bootstrapped variance estimates, such as standard deviation, standard error, confidence interval and residual standard error) for image replication levels above 30, but to provide a margin of error we analysed 35 images per plot. The power analysis was executed in R using package *plyr* [59] (see ESM Table S1 for more details). We also imposed a minimum distance of two meters between selected images to minimize spatial autocorrelation within each plot. All randomly selected images were uploaded into CoralNet (http://coralnet.ucsd.edu/http://coralnet.ucsd.edu/), an online repository and resource for benthic image analysis that facilitates annotation of benthic survey images [60]. Twenty-five points were randomly overlaid on each image, and the taxon or substratum underneath each point identified into one of 32 categories (Table S1). The proportional cover of each category in each quadrat was calculated as the number of points overlying that category divided by 25. Corals were identified to either genus or morphospecies (e.g. branching *Acropora*) because the resolution of images was not always sufficient to distinguish between species.

### Statistical analyses

Analysis of community composition was performed using multivariate techniques in PERMANOVA+ for PRIMER v6 [61]. Permutational Multivariate Analysis of Variance (PERMANOVA) [62] was used to examine changes in community composition among plots and years. To identify differences among plots within a single year we used the ‘Unrestricted permutations of raw data’, the recommended option for single-factor PERMANOVA [61]. To examine changes in community composition across years, we ran PERMANOVA with ‘year’ as a fixed factor and ‘plot’ as a random factor (to account for the initial variability among plots but focus the analysis on any consistent changes over the study period) using permutation of residuals under a reduced model [61]. Homogeneity of multivariate variance among years was examined using Permutational Analysis of Multivariate Dispersions (PERMDISP). All PERMANOVA analyses were performed on square-root transformed Bray-Curtis similarity matrices using Type III (partial) sums of squares with 9999 random permutations. Similarity Percentages (SIMPER) identified the contribution of the dominant taxonomic groups to the total variability between plots and years [63]. The relationship between all plot/year combinations was visualised using Principal Coordinates Analysis (PCO), with multiple partial correlation vectors indicating the relationship between the dominant taxa and sites.

We used generalized linear mixed-effects models (GLMM) in R (package *mass* R v3.0) [64] to compare changes in coral and macroalgal percent cover in each image and plot across years. Coral cover was modelled as a function of year using a linear mixed effect model. Year was designated as an ordered categorical explanatory variable with four levels (2010, 2011, 2012 and 2013). Macroalgal cover was modeled using two-part models with binomial (for presence absence) and negative binomial distributions. Both coral and algal cover were modeled hierarchically using plot as a random effect to account for variability among plots, and all models incorporated a linear autocorrelation term to account for temporal autocorrelation between years. The choice of model was informed by the need to account for random effects of plot, the need to account for over-dispersed data and potential autocorrelation. All initial models tested the interaction between year and plot, but the interaction term was removed as it was not significant. Models were simplified following the parsimony principle and no significant terms were removed from the final model. Assumptions of homoscedasticity, independence and non- autocorrelation were confirmed using residual plots of all models.

## Results

**Figure 2:**
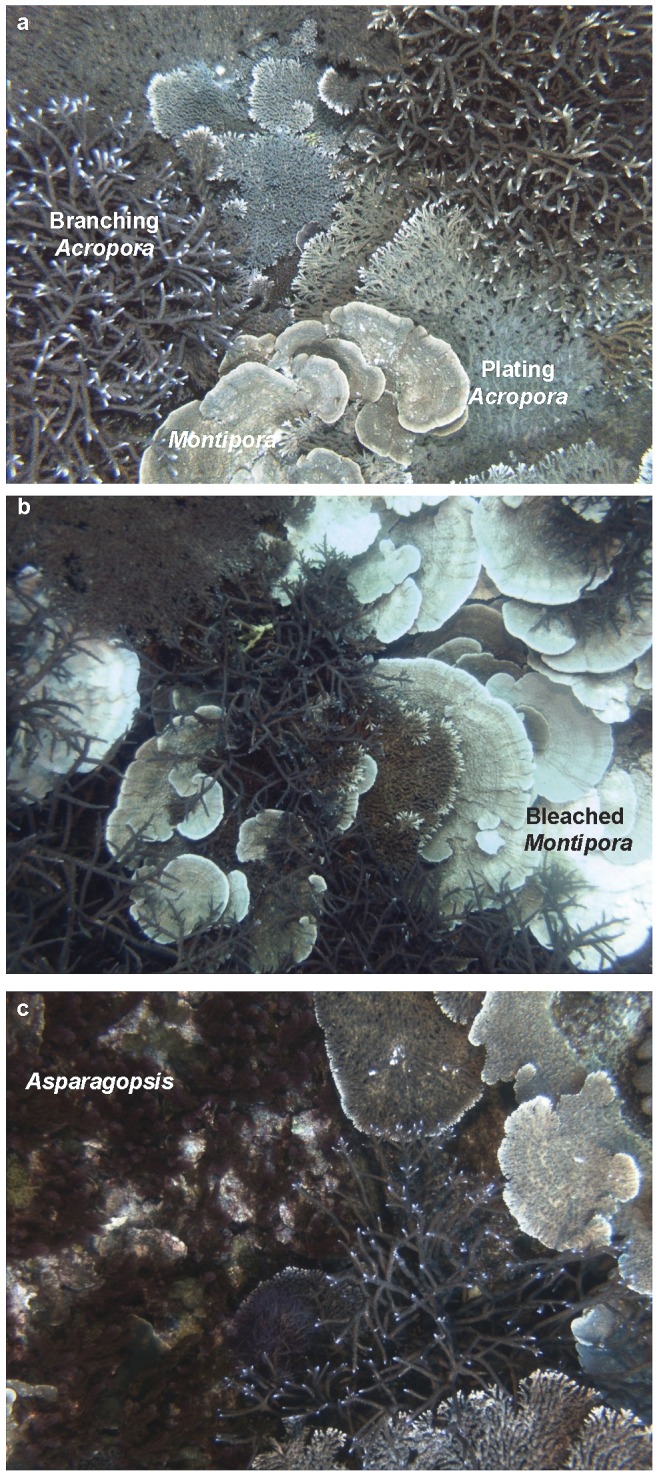
Autonomous Underwater Vehicle (AUV) images showing examples of shifts in community composition from 2010–2013; (a) high abundance of branching *Acropora*, plating *Acropora* and *Montipora* in 2010; (b) bleached *Montipora* adjacent to unbleached *Acropora* in 2011; (c) red macroalga *Asparagopsis* colonising substrate exposed by coral decline in 2013.

Prior to bleaching, benthic assemblages within the Geebank plots were dominated by hard corals, which occupied ∼73% of available the substrate. Ninety-five percent of the coral assemblage was composed of three coral morphospecies: branching *Acropora*, plating/tabular *Acropora* (plating *Acropora* hereafter), and plating *Montipora* (Fig. 2a). Macroalgae occupied ∼11% of the substrate, composed primarily of three taxa: the brown alga *Lobophora*, and the red algae *Asparagopsis* and *Sarcomenia*. Despite all three plots being separated by ∼100 m, community composition among plots was significantly different prior to bleaching (P = 0.001 for pairwise combinations in 2010). Plots 1 and 3 were both coral-dominated (coral cover >80%), although plot 1 had higher abundance of plating corals (*Montipora* in addition to plating *Acropora*), while branching *Acropora* was more abundant in Plot 3 (Table 1). In contrast, plot 2 had a lower abundance of plating corals, and was instead characterised by branching *Acropora* and the red macroalga *Sarcomenia*.

**Table 1:** Most abundant taxa in each plot in each year, identified using Similarity Percentages (SIMPER) analysis.

	Plot 1	Plot 2	Plot 3
**2010**	Plating *Acropora*	Branching *Acropora*	Plating *Acropora*
	*Montipora*	*Sarcomenia*	Branching *Acropora*
**2011**	Plating *Acropora*	*Lobophora*	Plating *Acropora*
	*Montipora*	Branching *Acropora*	Branching *Acropora*
	N/S	***	*
**2012**	Plating *Acropora*	*Lobophora*	Branching *Acropora*
	*Montipora*	Plating *Acropora*	Plating *Acropora*
	***	***	**
**2013**	Plating *Acropora*	*Lobophora*	Branching *Acropora*
	Branching *Acropora*	*Asparagopsis*	Plating *Acropora*
	**	***	***

* indicates significant change in community composition from 2010; * = 0.1, ** = 0.001, *** = 0.0001.

Bleaching was observed in 10.5% of corals in 2011, but the extent of bleaching varied substantially among plots and taxa. For corals, bleaching was most severe in *Montipora*, with bleached colonies regularly observed adjacent to unbleached *Acropora* in April 2011 (Fig. 2b). In total, 72% of plating *Montipora* were bleached during the 2011 survey, compared to only 4.5% of plating *Acropora* and 0.5% of branching *Acropora*. Bleaching was most prevalent in plot 1, with 21% of all coral colonies showing signs of bleaching, compared to 3% and 7% for plots 2 and 3, respectively. Some bleaching was also observed following the smaller thermal anomalies in 2012 and 2013, although the incidence of bleaching was lower than in 2011. In total, the proportion of corals showing signs of bleaching was 4.2% in 2012 and 1.6% of colonies in 2013, compared to 10.5% in 2011. No taxa or plots bleached as severely as *Montipora* in 2011 in any other year, although bleaching (generally partial bleaching) was observed in 19% of plating *Acropora* in plot 2 during 2012. In all other plot/year combinations, the incidence of bleaching was <8% for any single taxon. Macroalgae did not exhibit visible signs of bleaching. However, there were substantial changes in the relative abundance of each macroalgal taxon over the four-year study period. *Sarcomenia* and *Aspargopsis*, two of the most abundant macroalgae in 2010, were completely absent in 2011, while *Lobophora* increased from 2 to 11% of total benthic cover. *Asparagopsis* returned in 2012 and had increased significantly by 2013, but *Sarcomenia* remained rare.

**Figure 3:**
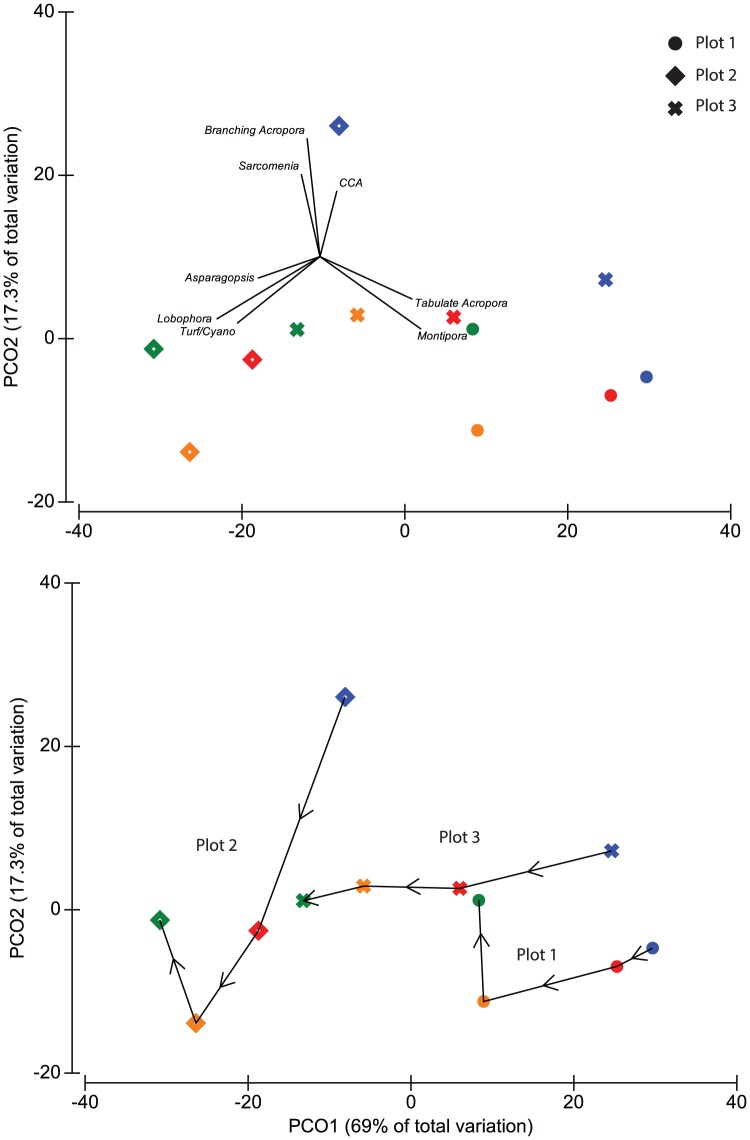
Principal coordinates plot of shifts in composition of dominant benthic taxa from 2010–2013. Shapes indicate the different plots (circles = plot 1, diamonds = plot 2 and crosses = plot 3, while colours indicate different years (blue = 2010, red = 2011, orange = 2012 and green 2013). A general trend of declines in plating corals and increased macroalgae were observed in all plots, although coral decline was most pronounced in plot 3. Declines in plating corals were offset by increases in branching *Acropora* in plots 1 and 2 between 2012 and 2013.

All three plots experienced significant changes in community composition over the study period (Table 1). However, there was substantial variability among plots in the magnitude and nature of these shifts. All three plots exhibited a general shift away from plating corals (*Montipora* and plating *Acropora*) (Fig. 3). Plots 1 and 3 were similar in composition in 2010, but showed very different trajectories from 2011–2013. In plot 1, declines in plating corals were offset by an increase in branching *Acropora*, while plot 3 exhibited a large increase in the abundance of macroalgae. Plot 2 supported lower coral and higher macroalgal abundance during the initial survey in 2010 and coral abundance continued to decline after 2011, apart from an increase in branching *Acropora* between 2012 and 2013. Among macroalgae, *Asparagopsis* increased in abundance from zero in 2011 to 14% of total benthic cover in 2013, where it was commonly observed growing on top of dead coral skeletons (Fig. 2c). In contrast, *Sarcomenia* remained rare in 2013, occupying just 1.5% of the substrate in 2013. *Lobophora* was less abundant in 2013 than immediately following the heatwave in 2011, but increased in abundance from 2% (in 2010) to 8.5% (in 2013) of benthic cover over the study period. The timing of community shifts also varied among plots: plot 1 showed no significant change in composition between 2010 and 2011, but had changed significantly by 2012 (p = 0.0001) (Table 1). In contrast, community shifts in plots 2 and 3 occurred more rapidly, with significant changes observed between 2010 and 2011. Despite these changes in composition, there was no significant difference in multivariate dispersion among years in any plot.

**Figure 4:**
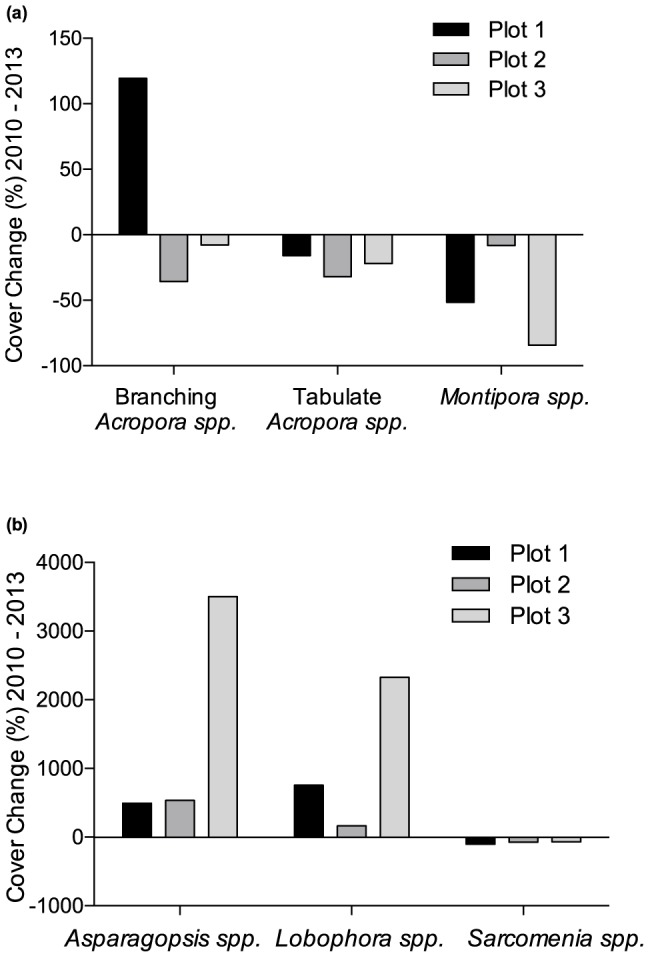
Changes in percent cover of hard coral (a) and macroalgae (b) in each plot from 2010 to 2013.

From 2010 to 2013 *Montipora* and plating *Acropora* had declined by 48% and 23%, respectively, across all plots compared to 2010, but branching *Acropora* increased by 25%. However, shifts in the abundance of different taxa were highly variable among plots (Fig. 4). Branching *Acropora* increased by 120% in plot 1, but declined 36% in plot 2 and 7% in plot 3 over the study period. The majority of increase in branching *Acropora* in plots 1 and 2 occurred between 2012 and 2013. Among macroalgae, *Asparagopsis* and *Lobophora* increased markedly in plot 3 over the study period, but not in plots 1 or 2.

### Change in hard coral and macroalgal relative abundance

**Figure 5:**
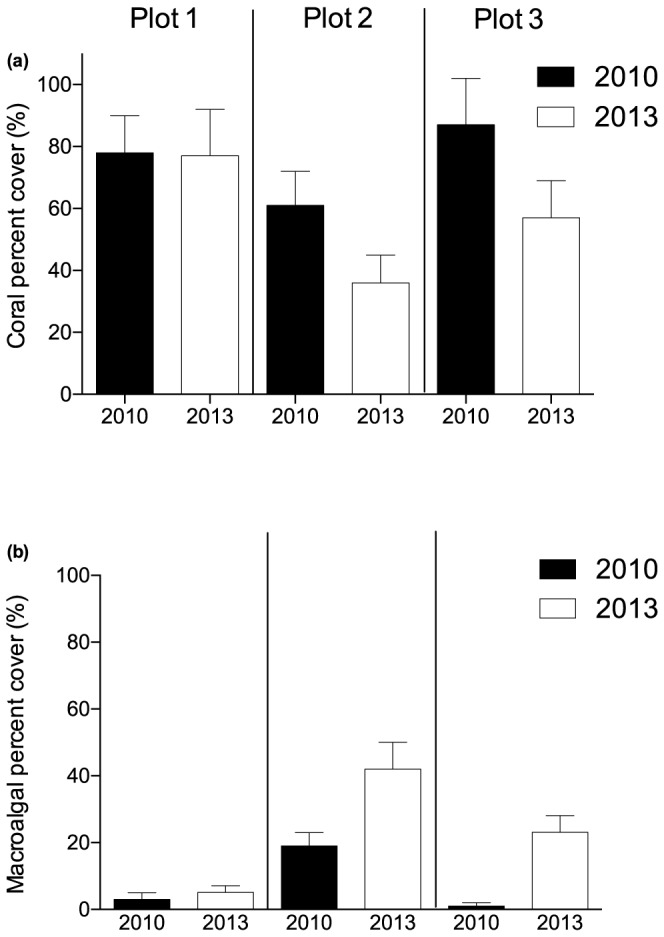
Changes in the abundance of the dominant coral and macroalgal taxa across the three plots from 2010–2013.

Hard corals declined in abundance from 73% to 59% of total benthic cover across all plots from 2010 to 2013, while macroalgae increased from 11% to 24%. Coral cover declined significantly by 8% from 2010 to 2011, then declined further by 7% from 2011 to 2012 and by 5% from 2012 to 2013 (Table 2). Prior to bleaching, plots 1 and 3 both supported high coral cover (>80%) and low macroalgal cover (≤5%); while, plot 2 had lower coral (55%) and higher macroalgal (24%) cover (Fig. 5). However, declines in total coral cover were also highly variable among plots (Table 2), and appeared unrelated to either the amount of pre-existing coral cover or the extent of bleaching.

**Table 2:** Summary statistics of the final GLMMs for coral percent cover (LMM) and algal percent cover (two-part model) from 2010–2013.

Coral cover	Coefficient	SE	p-value
2010	79.61	9.64	0.0000
2011	−8.35	3.80	0.0287
2012	−15.32	3.81	0.0001
2013	−20.35	3.81	0.0000
Random effect	Coefficient	Residual	
plot	15.963	26.439	

Algal cover	Presence/absence model		Negative binomial model	
	Coeff. (SE)	p-value	Coeff. (SE)	p-value
2010	−0.374 (0.756)	0.6210	2.931 (0.254)	0.0000
2011	0.055 (0.331)	0.8680	0.153 (0.168)	0.3610
2012	0.738 (0.328)	0.0245	0.171 (0.157)	0.2786
2013	1.355 (0.334)	0.0001	0.484 (0.154)	0.0017
Random effect	Coefficient	Residual	Coefficient	Residual
plot	1.547	1.344	0.141	0.948

SE = standard error, Coeff. = Coefficient.

Macroalgal cover in 2013 also remained low (6%) in plot 1, but increased from 24% to 43% and 5% to 24% of total benthic cover in plots 2 and 3, respectively (Fig. 5). In contrast to the declines observed in coral cover, macroalgal cover increased significantly between 2010 and 2013, but only by 0.5% across the three plots. Algal cover did not change significantly between 2010, 2011 and 2012. Interestingly, the presence of algae in any plot was more likely in 2012 and 2013 than in 2010 and 2011 (Table 2).

## Discussion

The high summer sea temperatures at the HAI had multifaceted impacts on the benthic community at Geebank, leading to significant shifts in the composition of benthic communities over the four-year study period. Two years after the 2011 heatwave, no plots showed trajectories indicative of recovery to 2010 composition, with plating corals and subtropical macroalgae appearing particularly susceptible to thermal stress. Despite being located in close proximity and in a similar geomorphic setting, the three plots showed differing post-bleaching trajectories. Plot 1 was able to maintain high coral cover due a shift from plating corals to branching *Acropora*. Plots 1 and 3 supported similar communities in 2010, but by 2013 plot 3 appeared on a trajectory from coral-dominated to algal-dominated states. In contrast, plot 2 exhibited substantially lower coral and higher macroalgal abundance prior to bleaching, and although composition changed significantly among all years algal cover did not increase significantly until 2013 in plot 2.

At the HAI, diver-based surveys indicated ∼22% of corals in shallow depths (6–9 m) bleached during the heatwave [26], while AUV surveys at 15 m depth estimated the incidence of bleaching to 4–19% [65]. Our study is among the first to examine post-bleaching trajectories of benthic assemblages on a high-latitude reef in Australia, and further demonstrates that high-latitude reefs are not immune from coral declines due to temperature stress. Many corals that showed negative responses to thermal stress were likely subtropical species (e.g. the plating *Acropora* species *A. stoddarti* and *A. spicifera* that dominate the assemblage at the HAI [66]) and therefore had little capacity to withstand a large-magnitude thermal anomaly, even though water temperatures of 27–29° were below the bleaching thresholds for many tropical reefs. These results suggest that even if sea temperatures remain within the known thermal tolerance limits for many tropical coral species, high-latitude reefs are likely to undergo significant shifts in community composition as subtropical species are displaced by tropical taxa.

Our results confirm previous studies showing bleaching response can be highly variable among taxa and across relatively small spatial scales [23], [32], [67]–[70]. Interestingly, at Geebank coral bleaching incidence during the 2011 heatwave showed little correlation with changes in coral cover. In 2011, the highest incidence of bleaching was observed in plot 1, but from 2011 to 2013 coral decline was greater in plots 2 and 3. These results highlight the importance of considering local-scale variability when assessing not only bleaching mortality, but also post-bleaching trajectories on coral reefs. However, identifying the cause of the variability observed at Geebank is difficult. Of the three dominant coral taxa, *Montipora* was clearly the most vulnerable to bleaching, with 72% of all colonies observed in 2011 bleached or recently dead, compared to only 4.5% of plating *Acropora* and 0.5% of branching *Acropora*. Plot 1 contained a greater abundance of *Montipora* in 2010 and therefore exhibited the highest incidence of bleaching. However, declines in *Montipora* in plot 1 were offset by a 120% increase in branching *Acropora* from 2011 to 2013. In contrast, macroalgae increased substantially in plots 2 and 3 while branching *Acropora* declined or remained stable (Fig. 4). The cause of such strong site-specific patterns is not clear, given that all three plots are located in relatively close proximity, in a similar geomorphic and gross oceanographic setting and at comparable depths.

Given our surveys were conducted annually, we cannot rule out additional undetected disturbance events contributing to the observed community shifts. Peak temperatures during the 2011 heatwave occurred in early March [24], and the absence of bleached or recently dead *Acropora* colonies in our survey six weeks later suggests that no additional mortality occurred as a direct result of the 2011 heatwave. However, there is evidence of some additional bleaching between 2011 and 2013. NOAA Coral Reef Watch indicates potential bleaching conditions did occur at the HAI in ∼1 month prior to our survey in 2012 (http://coralreefwatch.noaa.gov/satellite/vs/australia.php), and we did observe some bleaching in both 2012 and 2013. However, the incidence of bleaching was substantially lower than in 2011, and many colonies were partially bleached and/or showing evidence of disease (Fig. S1), causing difficulty in attributing declines to a single cause. Increased susceptibility to disease is a common result of sub-lethal thermal stress in corals, and is an important indirect cause of coral mortality following bleaching events [7], [70]–[73]. In addition to bleaching-induced mortality, some coral loss in Western Australia during 2011 was attributed to storm damage due to an unusually active cyclone season associated with the strong La Niña [26]. However, no storm passed within 400 km of the HAI, well beyond the 50–70 km range generally associated with cyclone damage [74], [75]. Furthermore, the AUV images showed no sign of broken corals or overturned plates characteristic of storm damage, despite their high mechanical vulnerability [76]. Consequently, we suggest that coral decline observed at Geebank from 2011 to 2013 was caused primarily by secondary effects of thermal stress (e.g. disease outbreaks or competition with macroalgae) and/or cumulative heat stress from successive years of high summer temperatures, rather than as a direct result of bleaching-induced mortality during 2011. Regardless of the cause, our results demonstrate that small-scale variability in habitat conditions can result in a mosaic of responses, highlighting the importance of examining post-bleaching trajectories and emphasising the need for a better understanding of local environmental drivers, especially on high latitude reefs.

Among macroalgae, the tropical taxa *Lobophora* and *Asparagopsis* increased over the study period but the temperate *Sarcomenia* became rare. Macroalgal occurrence can be dynamic and the abundance of different taxa may show strong seasonality [77], [78], complicating attempts to quantify variability among years. By collecting data during the same month (April) in each year, the shifts in macroalgal abundance reported here are likely to reflect actual changes rather than simply seasonal shifts. Differences in vulnerability among taxa were also consistent with expectations based on their geographic ranges. The HAI represent the northern, warm-edge range boundary for *Sarcomenia delesserioides*, a temperate species that occurs along the south coast of Australia, while *Asparagopsis taxiformis* and *Lobophora variegata* are widespread tropical species distributed throughout the Indo-Pacific [79]. Although both *Sarcomenia* and *Asparagopsis* declined substantially from 2010 to 2011, *Asparagopsis* increased significantly from 2011–2013, whereas *Sarcomenia* remained rare. Species with wider geographic ranges that encompass the tropics would be expected to cope better with warmer temperatures than subtropical species at their range boundary, supporting the hypothesis that the observed shifts in macroalgal abundance were likely due to temperature and not seasonality. These results suggest tropical macroalgae are well equipped to take advantage of temperature-induced disturbances on high-latitude reefs. Consequently, increases in the frequency of thermal anomalies may fundamentally change the composition of macroalgal assemblages. In addition, our results also suggest that benthic communities in the HAI, and potentially other high-latitude reefs, may respond differently to thermal stress compared to Indo-Pacific reefs at lower latitudes. Although there is extensive literature on disturbance-induced coral-algal shifts on coral reefs [80], [81], such shifts are uncommon in the Indo-Pacific, where macroalgae dominate only 1% of reefs [82], [83]. Macroalgal shifts are more common on western Atlantic reefs, potentially due to factors including higher rates of macroalgal growth and/or recruitment and lower herbivore biomass and diversity [83]. Many of these conditions prevail on high-latitude reefs, suggesting they may be less resistant to macroalgal shifts than their counterparts at lower latitudes.

Post-disturbance trajectories in benthic community composition are highly variable among events and regions [7], [84], therefore predicting longer-term trajectories of benthic communities in the HAI is difficult. Some reefs recover rapidly after bleaching-induced mortality, whereas others may show little recovery many years after a bleaching event [84]–[86]. Why some reefs recover while others do not is not always clear, but the absence of additional or chronic stressors (e.g. overgrowth of hard substrate by macroalgae, outbreaks of *Acanthaster planci* and coral disease) is clearly important for coral recovery [7], [33], [86]. Recovery rates on high-latitude reefs are poorly known, but the lower coral recruitment and greater competition with macroalgae may slow recovery in the HAI compared to reefs at lower latitudes [39]. The frequency and severity of thermal anomalies in HAI in recent years is unprecedented in at least two centuries [52], increasing the likelihood that benthic communities will experience significant changes in coming decades. Tropical-temperate transition regions influenced by a strong poleward boundary current such as the HAI provide a model system to detect the biotic tropicalization of temperate latitudes [17], [20], [50]. Therefore, we recommend ongoing monitoring of coral reefs in the HAI to identify long-term trajectories of benthic community composition on high-latitude reefs, which will provide important insights into the nature of community reassembly in response to climate change. More broadly, our results support the hypothesis that stochastic, extreme events may be more important than climatic means for determining the effects of climate change on ecological communities, and that stochastic events may cause and/or accelerate sustained shifts in species composition and abundance [44], [87], [88]. Understanding the ecological consequences of changes in the frequency and/or severity of acute disturbances is therefore critical for predicting the effects of climate change on coral reef ecosystems, and such predictions should include both tropical and subtropical ecosystems.

## Supporting Information

Figure S1 **AUV image from 2013 showing partial mortality characteristic of white-band disease on an** ***Acropora*** **colony at Geebank.**
(TIFF)

Table S1 **Power analyses summary table showing the residual standard error (%) for each class and different no. of images analysed (n) from 5 to 50.**
(DOCX)
