# Supplementary figures and images for: Variable Responses of Benthic Communities to Anomalously Warm Sea Temperatures on a High-Latitude Coral Reef
Source: PLoS One. 2014 Nov 26;9(11):e113079. doi: 10.1371/journal.pone.0113079 (PMC4245080; doi:10.1371/journal.pone.0113079)

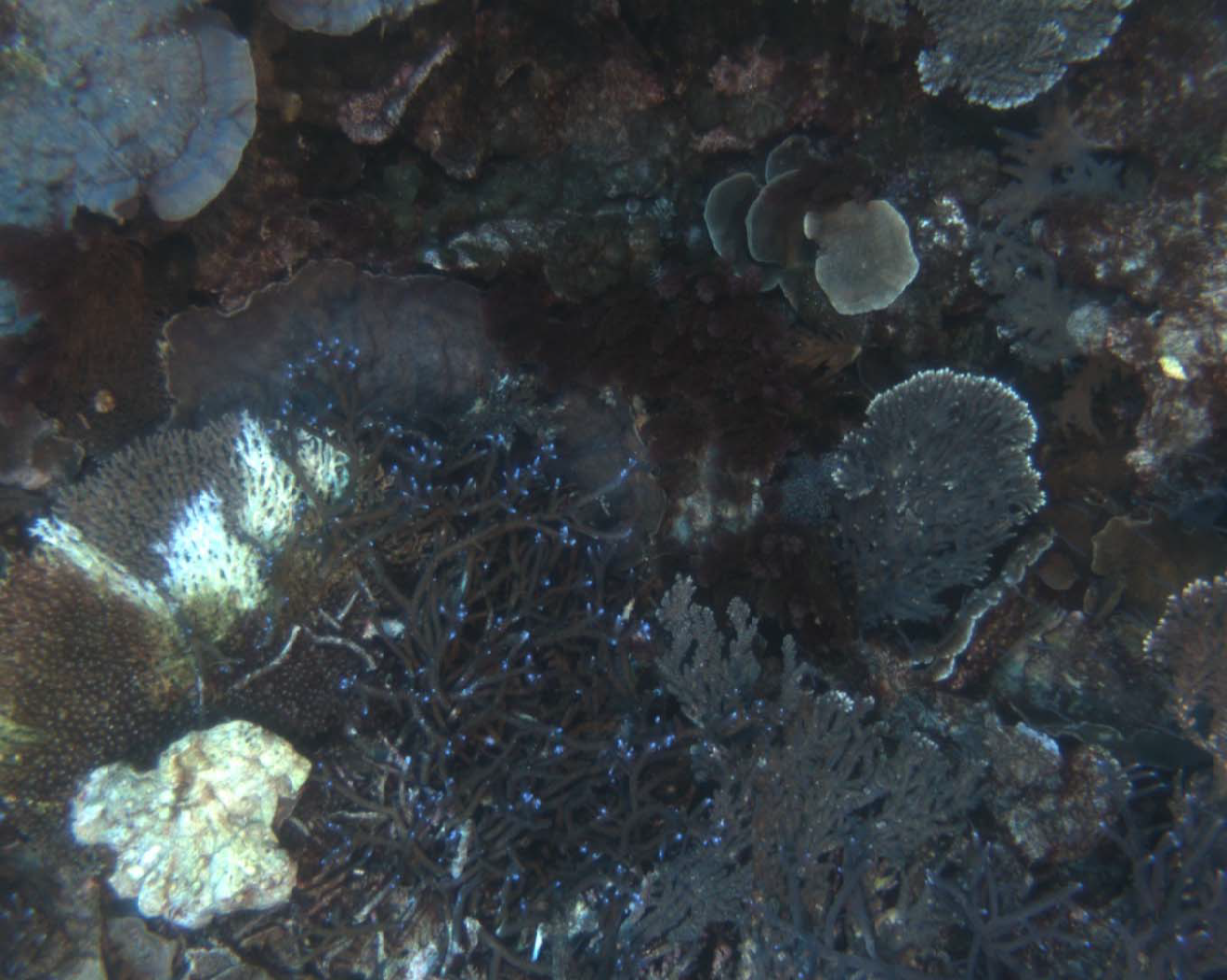

Supplement: Figure S1 — AUV image from 2013 showing partial mortality characteristic of white-band disease on an Acropora colony at Geebank. (TIFF) [file pone.0113079.s001.tiff]
