# Supplementary material for: Variable Responses of Benthic Communities to Anomalously Warm Sea Temperatures on a High-Latitude Coral Reef
Source: PLoS One. 2014 Nov 26;9(11):e113079. doi: 10.1371/journal.pone.0113079 (PMC4245080; doi:10.1371/journal.pone.0113079)
Supplement: Table S1 — Power analyses summary table showing the residual standard error (%) for each class and different no. of images analysed (n) from 5 to 50. (DOCX) [file pone.0113079.s002.docx]

| n | 50 | 45 | 40 | 35 | 30 | 25 | 20 | 15 | 10 | 5 |
| --- | --- | --- | --- | --- | --- | --- | --- | --- | --- | --- |
| *Acropora* branching | 0.009 | 0.00973 | 0.01015 | 0.01055 | 0.0114 | 0.01256 | 0.0138 | 0.01614 | 0.02016 | 0.02954 |
| *Acropora* tabulate | 0.00752 | 0.00813 | 0.00813 | 0.00875 | 0.00966 | 0.01001 | 0.01172 | 0.01346 | 0.01661 | 0.02477 |
| *Acropora* corymbose | 0.09774 | 0.10755 | 0.10906 | 0.1142 | 0.12529 | 0.13887 | 0.16387 | 0.17348 | 0.21353 | 0.26782 |
| Algae | 0.02065 | 0.0221 | 0.02233 | 0.02528 | 0.02574 | 0.02866 | 0.03213 | 0.03701 | 0.04765 | 0.06425 |
| *Asparagopsis spp.* | 0.02473 | 0.0268 | 0.02687 | 0.02941 | 0.03208 | 0.03471 | 0.03825 | 0.04674 | 0.05593 | 0.07713 |
| *Acropora* branching bleached | 0.0621 | 0.06155 | 0.06652 | 0.07098 | 0.07739 | 0.087 | 0.0972 | 0.11188 | 0.1338 | 0.20333 |
| *Acropora* tabulate bleached | 0.08162 | 0.08148 | 0.08911 | 0.09361 | 0.10216 | 0.10496 | 0.12857 | 0.14077 | 0.17561 | 0.24946 |
| *Montipora* bleached | 0.10148 | 0.10104 | 0.11197 | 0.11853 | 0.12528 | 0.13862 | 0.15686 | 0.19819 | 0.24736 | 0.31507 |
| CCA | 0.01076 | 0.01189 | 0.01245 | 0.013 | 0.01404 | 0.01605 | 0.01745 | 0.01987 | 0.02471 | 0.03384 |
| *Codium spp.* | 0.07245 | 0.07482 | 0.08319 | 0.08984 | 0.09553 | 0.10145 | 0.1182 | 0.12973 | 0.15707 | 0.23035 |
| Cyanobacteria | 0.04367 | 0.04631 | 0.04912 | 0.05204 | 0.05542 | 0.06295 | 0.06966 | 0.08115 | 0.09393 | 0.13729 |
| Dead Coral | 0.07315 | 0.07453 | 0.07671 | 0.08293 | 0.08732 | 0.09794 | 0.11267 | 0.13161 | 0.16634 | 0.22338 |
| *Echinophyllia spp.* | 0.03779 | 0.03954 | 0.04142 | 0.04375 | 0.04774 | 0.05192 | 0.0571 | 0.06938 | 0.08225 | 0.11632 |
| *Favites spp.* | 0.10104 | 0.1031 | 0.11246 | 0.12289 | 0.13602 | 0.14167 | 0.17132 | 0.18725 | 0.20922 | 0.30257 |
| *Goniopora spp.* | 0.04616 | 0.0483 | 0.05216 | 0.05559 | 0.05876 | 0.06716 | 0.07339 | 0.08751 | 0.10477 | 0.15044 |
| *Lobophora spp.* | 0.02326 | 0.02495 | 0.02747 | 0.02651 | 0.03081 | 0.03386 | 0.03616 | 0.04413 | 0.05399 | 0.07416 |
| *Millepora spp.* | 0.09792 | 0.10163 | 0.10988 | 0.12241 | 0.12803 | 0.13978 | 0.16019 | 0.1795 | 0.22563 | 0.31697 |
| *Montipora spp.* | 0.01121 | 0.01158 | 0.01217 | 0.01346 | 0.01452 | 0.01516 | 0.01751 | 0.02035 | 0.02449 | 0.03462 |
| *Mycedium spp.* | 0.07249 | 0.07346 | 0.08106 | 0.08666 | 0.09602 | 0.10619 | 0.12014 | 0.131 | 0.16327 | 0.23041 |
| Other Hard corals | 0.04444 | 0.04771 | 0.04905 | 0.05489 | 0.05803 | 0.06237 | 0.07243 | 0.07637 | 0.09542 | 0.13406 |
| *Pectiniid spp.* | 0.02626 | 0.02792 | 0.03117 | 0.03283 | 0.03478 | 0.03688 | 0.0449 | 0.04877 | 0.06274 | 0.09004 |
| *Platygyra spp.* | 0.0774 | 0.08018 | 0.08792 | 0.09314 | 0.09374 | 0.11045 | 0.12288 | 0.14723 | 0.17774 | 0.24862 |
| *Porites spp.* | 0.06248 | 0.06898 | 0.07594 | 0.07383 | 0.08671 | 0.08887 | 0.09589 | 0.11402 | 0.14261 | 0.19825 |
| Sand | 0.09737 | 0.10342 | 0.10902 | 0.11906 | 0.12669 | 0.13761 | 0.14704 | 0.18742 | 0.20871 | 0.29602 |
| *Sarcomenia spp.* | 0.01485 | 0.01534 | 0.01642 | 0.01721 | 0.01955 | 0.02068 | 0.02271 | 0.02702 | 0.03215 | 0.047 |
| Turf algae | 0.01582 | 0.0167 | 0.01657 | 0.01849 | 0.02077 | 0.02165 | 0.02469 | 0.02838 | 0.03328 | 0.04999 |
| Unclear | 0.00945 | 0.00987 | 0.01083 | 0.01129 | 0.01242 | 0.0139 | 0.01507 | 0.01776 | 0.02145 | 0.03091 |
